# Derivation of HLA types from shotgun sequence datasets

**DOI:** 10.1186/gm396

**Published:** 2012-12-10

**Authors:** René L Warren, Gina Choe, Douglas J Freeman, Mauro Castellarin, Sarah Munro, Richard Moore, Robert A Holt

**Affiliations:** 1 BC Cancer Agency, Michael Smith Genome Sciences Centre, Vancouver, British Columbia V5Z 1L3, Canada; 2 Department of Molecular Biology and Biochemistry, Simon Fraser University, Burnaby, British Columbia V5A 1S6, Canada

## Abstract

The human leukocyte antigen (HLA) is key to many aspects of human physiology and medicine. All current sequence-based HLA typing methodologies are targeted approaches requiring the amplification of specific HLA gene segments. Whole genome, exome and transcriptome shotgun sequencing can generate prodigious data but due to the complexity of HLA loci these data have not been immediately informative regarding HLA genotype. We describe HLAminer, a computational method for identifying HLA alleles directly from shotgun sequence datasets (http://www.bcgsc.ca/platform/bioinfo/software/hlaminer). This approach circumvents the additional time and cost of generating HLA-specific data and capitalizes on the increasing accessibility and affordability of massively parallel sequencing.

## Background

Due to its central role in adaptive immunity, human leukocyte antigen (HLA) is implicated in wide ranging areas of medicine, from infectious disease and vaccinology to cancer, autoimmunity, aging and regenerative and transplantation medicine [1-7]. The HLA locus is the most polymorphic region of the genome with over 5,000 variant HLA-class I allelic sequences catalogued to date. This genetic heterogeneity is the principal challenge to HLA typing methodologies, and it is the reason why this region has remained largely opaque to analysis by next-generation sequencing (NGS) platforms. Conventional sequence-based HLA typing approaches, the most recent of which exploits the sequence throughput of the Illumina MiSeq [8] and relatively long sequence reads of the 454 NGS platform [9], are targeted assays that rely on amplification of hypervariable sub-regions of these loci and variant detection within these amplicons. As such, HLA calls are based on sequence information that is not as comprehensive as for shotgun datasets, and must be generated *de novo *for each subject. The widespread uptake of large-scale genome, exome and transcriptome shotgun sequencing approaches for biomedical research, and now for clinical use, prompted us to explore the utility of these types of NGS data sets for HLA typing. The need has been for a solution to the problem of managing the many millions of short sequence reads NGS technologies produce, managing the many thousands of reference allele sequences, and integrating all of these data in a manner that maximally informs HLA content. Here we present a method for HLA allele prediction from next-generation shotgun sequence datasets. We focus on data generated from the Illumina platform, from which most sequence data are currently derived worldwide. Importantly, HLA allele assignments from shotgun datasets can not be derived from standard alignment-based interpretive methods for the simple reason that the extant genome reference sequences [10,11] on which these methods rely do not provide any useful representation of HLA allelic diversity. Therefore, we have developed a computational pipeline that derives HLA allele predictions by targeted assembly of shotgun sequence data and comparison to a database of reference allele sequences. Our solution allows, for the first time, application of the power of NGS to the interrogation of one of the most important and complex sets of human genes. Our method is scalable, such that it will provide utility in extracting HLA information even from very large sequence data sets, such as those currently being compiled by various international consortia [12-15].

## Materials and methods

### Library construction and sequencing

Written informed consent was obtained from all donors and samples were collected following assessment of tissue specimens by a pathologist according to standardized operating procedures, immediately following surgical resection. Library construction and Illumina sequencing were performed as previously described for RNA-Seq [16] and whole genome shotgun (WGS) [17]. For the colorectal cancer (CRC) RNA-Seq study, four lanes of 100-nucleotide paired-end sequences were obtained for each of the two pools, providing an average of 5 million paired reads per sample. For WGS, approximately 430 million paired 100-nucleotide WGS reads (approximately 30× depth coverage human genome) from normal and tumor samples from four diffuse large B cell lymphoma patients were processed [17]. The sequencing data from the CRC study have been submitted to the NCBI Sequence Read Archive [18] under accession number SRP010181. A file describing the sample libraries is available at [19].

Exome capture libraries were prepared using the SureSelect system (Agilent) according to the manufacturer's instructions. Approximately 30 million (normal samples) and 120 million (normal plus tumor samples) 100-nucleotide exon capture paired-end sequence reads were generated from three ovarian cancer patients whose HLA alleles were verified by PCR-based methods. Verification of HLA allele predictions was accomplished by PCR amplification of exons 2 and 3 from HLA-I A, B and C, followed by capillary sequencing as previously described [20].

### IMGT/HLA sequences

HLA coding DNA sequence (CDS) and genomic sequence databases from release 3.3.0 and 3.4.0 were obtained, respectively, from [21]. HLA-I exon 2 and 3 concatenated sequence FASTA files were prepared using exon coordinates available from the flat file database (EMBL format) released by IMGT [22]. For HLA allele predictions from RNA-Seq data, we used concatenated exons 2 and 3 as sequence targets for assembly using the TASR assembly tool [23]. For predictions from genome and exome NGS data, we used HLA-I genomic sequences from major genes A, B and C.

### Computational HLA allele predictions by targeted read assembly

HLA CDS or genomic sequences from IMGT/HLA (sequence targets) are read by TASR (default options used with -i 1), creating a hash table of every possible 15-nucleotide word (k-mers) encountered. NGS data sets are interrogated for the presence of one of these k-mers in 5' (on either strand) and candidate reads recruited. Recruited reads seed the assembly in a manner analogous to that of SSAKE [24]. Only sequence contigs equal to or larger than a user-determined length (200 nucleotides chosen for this study) are considered for further analysis. Reciprocal BLAST [25] (v.2.2.22 with options -a 8 -F F -p blastn -m 7) alignments are performed between the contig and HLA CDS or genomic sequence databases depending on the read source (RNA-Seq or WGS and exon capture), parsed at runtime using PERL Bio::SearchIO modules and summarized. HLAminer parses these alignment files and generates a score and probability for each putative HLA coding variant identified from sequence contigs. Briefly, for each assembled contig, best BLAST HLA alignments are reported, tracking the sequence identity over the alignment portion, as well as over the length of the contig. Contigs are organized by increasing number of HLA sequences they co-characterize best, listing all possible *ex-equo *best hits and tracking HLA sequences that, reciprocally, best identify each contig. For each putative HLA, a score *S_HLA _*is calculated as the sum of score computed for each contig aligning to it. Individual contig scores factor in the contig depth of coverage, length and percent sequence identity, such that a score reflects the number of bases aligned to a particular HLA allele. A reciprocal best hit where a given HLA aligns best to a given contig doubles the score for the identified HLA sequence:

$$S_{HLA}=\overset{n}{\underset{Contig=1}{\sum }}Score_{Contig}=size^{*}depth^{*}\%sequence\text{\_}identity$$

For any given contig, the probability of characterizing a single HLA allele by chance is equal to the inverse proportion of HLA sequences in the sequence database. And since shorter contigs may not capture sufficient bases to characterize any one type unambiguously, the probability *P *that a contig characterizes one or another HLA type is mutually exclusive such that:

$$P_{Contig_{1}\text{\_}is\text{\_}HLA_{x}}=\sum P_{HLA}$$

The expect value (Eval) of each computationally determined HLA*x*, Eval_HLA*x*_, is calculated as:

$$Eval_{HLA_{x}}={\left({P_{Contig_{1}\text{\_}is\text{\_}HLA_{x}}}^{*}P_{HLA_{x}\text{\_}is\text{\_}Contig_{1}}\right)}^{*}{\left({P_{Contig_{2}\text{\_}is\text{\_}HLAx}}^{*}P_{HLA_{x}\text{\_}is\text{\_}Contig_{2}}\right)}^{*}\ldots^{*}\left({P_{Contig_{x}\text{\_}is\text{\_}HLA_{x}}}^{*}P_{HLA_{x}\text{\_}is\text{\_}Contig_{x}}\right)$$

since individual contig probabilities and reciprocal best hits are independent events. A short list of HLA allele groups (for example, A*02) and protein coding alleles (for example, A*02:01), sorted by decreasing score, are catalogued for each major HLA gene. When separate contigs characterize the same types, only the types that overlap are reported, unless the non-overlapping ones are characterized by additional, distinct contig(s). In addition, we summarize ambiguous HLA alleles using the P designation, when applicable.

### Simulated data sets

In separate experiments, we removed HLA CDS, exonic regions and genes from 15K randomly selected Ensembl [26] transcripts, approximately 220K exon regions [27] (SureSelect Target Enrichment, Agilent Technologies, Inc. [28] and the HuRef genome [11]). For each data type, we randomly generated 20 sets of six (2 × A, 2 × B, 2 × C) HLA-I alleles (total of 120 alleles). In triplicate experiments, we merged each set of six sequences with HLA-less CDS, exon regions or HuRef, respectively, and simulated at various depth of coverage 50-, 75-, 100- or 150-nucleotide paired-end reads with 0.5, 1, 2 or 3 error using SAMtools [29] wgsim, and ran TASR and BLASTN as described above. For the simulation from direct read pair alignments, we used the simulated reads described above and ran BWA [30] with defaults and generated HLAminer predictions from SAM files.

### Assessment metrics

We define the sensitivity as a proportion, that is, the number of HLA allele groups or protein coding alleles detected over the sum of distinct groups or protein coding alleles randomly chosen for the simulation or predicted by PCR, when applicable. The ambiguity rate is the proportion of all ambiguous predictions per total allele groups or protein coding alleles predicted. Ambiguous predictions arise when HLA allele groups or protein coding gene names differ despite having an identical score and probability. The specificity is defined as a proportion of number of groups or alleles predicted accurately divided by the total number of groups or alleles detected, respectively.

### HLA typing

HLA class I alleles were predicted directly from the RNA-Seq data as described [20]. Briefly, genomic DNA was extracted from patient granulocytes, and exons two and three from HLA class I genes (A, B, and Cw) were amplified by PCR [31]. PCR amplicons were cloned and sequenced using an ABI 3730XL instrument, according to standard procedures. Clone sequences were assembled using Phred/Phrap/Consed [32]. The resulting sequence data were aligned against all available exon 2 and 3 nucleotide sequences from the 3.1.0 release of the IMGT/HLA database [22] using ClustalW [33]. Protein coding allele assignments [34] were based on high-quality exact or synonymous matches at informative nucleotide positions.

## Results and discussion

**Figure 1:**
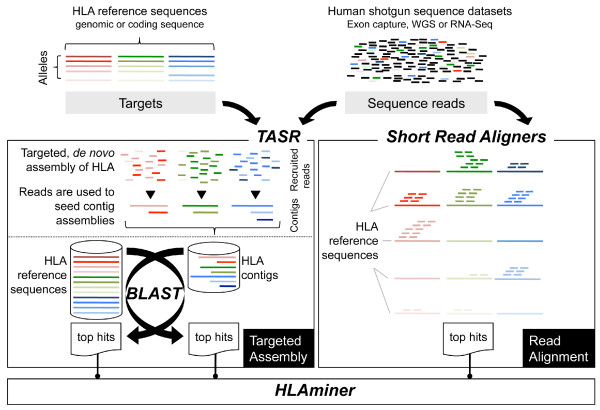
**Computational predictions of HLA-I from shotgun data by targeted assembly (left) or read alignment (right)**. For targeted assembly, NGS reads having their first fifteen 5' bases matching one of HLA CDS (RNA-Seq) or genomic (WGS/exon capture) sequences are recruited and assembled *de novo *with TASR. Resulting sequence contigs are aligned against a database sequence of all predicted HLA CDS (RNA-Seq) or genomic sequences (WGS/exon capture), tracking best HLA hit(s). Reciprocal best alignments are considered in the same manner. Putative allele assignments from shotgun datasets (HLAminer) are informed by contig length, depth of coverage and similarity to reference sequences, when applicable. The probability of each prediction being correct is estimated by determining the probability of that prediction being observed by chance.

To maximize the performance of HLAminer with short read data, we implemented stringent, localized, *de novo *assembly of sequence reads prior to alignment (Figure 1, left). Direct alignment of reads to reference alleles is also supported (Figure 1, right), but at the present time we find this modality has modest utility due to current limitations on read length. HLAminer predictions are arranged by HLA gene (for example, HLA class I A, B and C) and for each, putative alleles are ranked by highest scoring HLA protein coding alleles. A confidence value reflects the likelihood of each prediction (expect value) on a log10 scale. A sample output from HLAminer is shown (Table 1).

**Table 1:** Output from HLAminer HLA class I predictions from a CRC patient 100-nucleotide RNA-Seq sample

Allele^a^	Score^b^	Expect value (Eval)	Confidence (-10 × log10(Eval))
**HLA-A^c^**			
Prediction^d ^1 - A*02			
A*02:01P	64038.03	1.63E-06	57.9
Prediction 2 - A*11			
A*11:01P	5463.99	5.30E-09	82.8
			
**HLA-B**			
Prediction 1 - B*27			
B*27:05P	64579.61	2.67E-18	175.7
Prediction 2 - B*07			
B*07:02P	56662.08	6.63E-12	111.8
			
**HLA-C**			
Prediction 1 - C*07			
C*07:02P	49419.33	5.23E-08	72.8
Prediction 2 - C*02			
C*02:02P^e^	20466.00	6.64E-16	151.8
C*02:21^e^	20466.00	6.64E-16	151.8

^a^HLA protein coding alleles validated by PCR are shown in bold face. ^b^The protein coding allele predictions are arranged by decreasing score from most to less likely. ^c^Most likely HLA class I allele groups and protein coding alleles (Confidence (-10 × log10(Eval)) ≥ 20 Score ≥ 1,000) for each gene. ^d^The prediction rank factors in the maximum score for each predicted allele. ^e^Ambiguity arises when two or more HLA allele group or protein coding alleles have the same score (for example, C*02:02P and C*02:21).

For initial evaluation of HLAminer we relied on simulated data sets, which allowed us to determine the influence on performance of sequencing parameters such as depth of coverage, sequence read length, and sequence error. We produced simulated data sets for each of the three formats, RNA-Seq, WGS and exome, by taking reference sequences (Ensembl transcripts, the HuRef genome, and hg19 exon capture regions, respectively) and substituting HLA-I A, B and C sequences with two randomly chosen alleles of HLA-I A, B and C. From these modified references we generated faux sequence reads. For each sequence format, 20 such data sets were generated and these were queried in triplicate, yielding a total of 360 allele predictions per condition tested. The sensitivity, specificity and ambiguity of HLA class I allele prediction was evaluated by comparing the highest-scoring HLAminer predictions to the randomly selected alleles. By ambiguity we mean the prediction of multiple, equally probably alleles.

**Figure 2:**
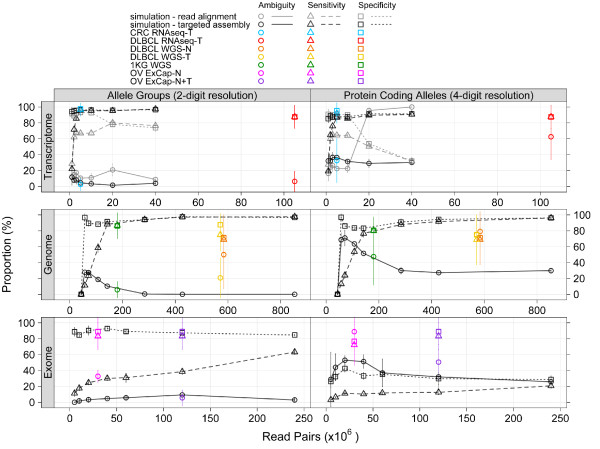
**HLAminer performance**. HLA allele group and protein coding allele predictions derived from targeted read assembly (black symbols) or direct read alignment (grey symbols) of simulated 100-nucleotide RNA-Seq, WGS and exon capture (ExCap) datasets were compared to original, spiked-in, HLA sequences and performance metrics evaluated (ambiguity, sensitivity and specificity represented by circle, triangle and square symbols, respectively). HLAminer predictions were also obtained from targeted assembly of colorectal cancer (CRC; blue symbols), lymphoma (DLBCL; red, orange and yellow symbols), 1000 Genomes (1KG; green symbols) and ovarian cancer (OV; violet and magenta symbols) patient tumor (T) and/or matched normal (N) shotgun datasets and compared to PCR-based HLA types to calculate performance metrics.

HLA nomenclature (for example, HLA A*02:01) defines the digits immediately following the asterisk as the allele group (two-digit resolution, formerly referred to as supertype) and the next set of digits (those following the semicolon, often referred to as four-digit resolution) as the individual protein coding allele [34]. Further separators and digits are sometimes used to describe allelic variants that contain silent nucleotide differences. Using simulated data, we found that at the level of HLA allele groups, RNA-Seq data provided high sensitivity and specificity (each >95.7%) with a low ambiguity (<4.5%), even at relatively low coverage (<5 million total read pairs) (Figure 2; Additional file 1). Likewise, WGS provided high sensitivity and specificity (each >97.3%) and no observable ambiguity (0.0%) for prediction of allele groups, but required substantially higher sequence depth, on the order of 400 million paired reads, to achieve this (Figure 2; Additional file 1). This is the equivalent of approximately 30× genome coverage with 100-nucleotide reads. For both RNA-Seq and WGS data, predictions at the level of individual protein coding alleles showed very similar sensitivity and specificity to that observed for allele group predictions, but ambiguity levels increased to approximately 30% (Figure 2; Additional file 1). Our expectations for HLA allele prediction from exome data were low, because allelic diversity of HLA coding sequence tends to have limited representation in standard capture reagents. For example, the Agilent SureSelect system that we use at our center contains 36 120-nucleotide RNA probes targeting the HLA class I region of hg19. Still, we included evaluation of this data type for the purpose of completeness, and with the understanding that a variety of HLA alleles could possibly be captured by imperfectly matching probes of this length. Our simulations revealed that exome data did in fact show some modest utility for HLA prediction, at least at the allele group level. For allele group prediction, high specificity (92.8%) and low ambiguity (4.7%) could be achieved at low coverage (40 million read pairs); however, considerably higher coverage was necessary to increase sensitivity, and even at very high exome coverage (240 million read pairs) sensitivity never approached that observed for the other data types. By comparison, for RNA-Seq, 5 million and 3 million 100-nucleotide RNA-Seq read pairs are required for 95% sensitivity and specificity, respectively. For WGS, 427 million and 57 million 100-nucleotide read pairs are needed for 95% sensitivity and specificity, respectively. Under the conditions tested, exome data did not provide such high levels of detection and prediction accuracy at any read depth and performance for predicting individual protein coding alleles from exome data was uniformly poor (Figure 2).

Overall, from simulation, RNA-Seq datasets provided the greatest utility for HLA prediction. This may be due, in part, to lower representation in RNA-Seq data of off-target regions, such as the minor HLA class I genes, pseudogenes, and HLA class II genes, compared to genome or exome data, where these regions would be expected to have approximately equal representation as the class I alleles of interest, A, B and C. The stark contrast in HLAminer predictions derived from RNA-Seq compared to WGS or exome capture highlights intrinsic properties of these datasets and their value for computational HLA predictions. Functional HLA-I alleles are expressed on all nucleated cells, and despite possible amplification biases in the RNA-Seq library construction protocol, the high abundance of HLA-I transcripts is such that relatively low depth of sequencing is needed for robust predictions (approximately 5 million). On the other hand, non-functional (null) HLA alleles that are present in the genome (but transcriptionally silent) can confound HLA prediction from WGS or exome capture data, since the functional alleles and null alleles are equally represented in these data types. HLAminer has the functionality to report predictions from null alleles, if desired.

We explored further the effects of read length (up to 150 nucleotides) and sequencing errors (up to 3%) with RNA-Seq data. Not unexpectedly, performance improved with increasing read length and decreasing base error (Table 2). Reads with length less than 75 nucleotides and error rates higher than 1% significantly impacted performance for prediction of individual protein coding alleles, but prediction of allele groups remained robust (Table 2).

**Table 2:** Effect of read length and base error on HLAminer predictions from targeted assembly of simulated RNA-Seq data^a^

HLA allele resolution	Base error (%)	Read length (nucleotides)	Sensitivity (mean ± SD%)	Specificity (mean ± SD%)	Ambiguity(mean ± SD%)
Two-digit	1.0	50	13.62 ± 2.80	92.86 ± 10.10	19.06 ± 16.53
		75	62.32 ± 3.62	90.27 ± 3.28	8.93 ± 2.67
		100	95.72 ± 0.53	96.31 ± 0.02	4.46 ± 3.84
		150	97.97 ± 2.80	95.73 ± 3.63	0.00 ± 0.00
Two-digit	0.5	100	93.04 ± 4.60	96.39 ± 1.44	2.91 ± 1.69
	1.0		95.72 ± 0.53	96.31 ± 0.02	4.46 ± 3.84
	2.0		64.64 ± 4.79	96.13 ± 1.43	13.40 ± 3.02
	3.0		6.67 ± 2.51	100.00 ± 0.00	8.59 ± 8.34
Four-digit	1.0	50	7.78 ± 1.92	60.51 ± 20.02	27.78 ± 4.81
		75	51.94 ± 2.93	77.36 ± 5.44	37.38 ± 11.16
		100	86.84 ± 1.75	88.13 ± 1.41	36.32 ± 4.76
		150	93.33 ± 3.63	93.07 ± 2.91	22.87 ± 2.40
Four-digit	0.5	100	84.72 ± 5.42	89.65 ± 2.25	24.03 ± 3.00
	1.0		86.84 ± 1.75	88.13 ± 1.41	36.32 ± 4.76
	2.0		56.94 ± 1.73	87.49 ± 4.77	39.14 ± 5.73
	3.0		4.44 ± 2.10	68.69 ± 17.03	37.22 ± 25.62

^a^In triplicate experiments, 5 million read pairs 50, 75, 100 or 150 nucleotides in length (top) and 100-nucleotide read pairs having 0.5, 1, 2 or 3% errors (bottom) were randomly generated from 20 sets of transcripts, each containing 6 randomly chosen reference HLA alleles. HLAminer predictions derived from targeted read assembly were compared to each reference set and the performance of HLAminer was assessed by measuring the specificity, sensitivity and ambiguity. SD, standard deviation.

Next, we evaluated the performance of HLAminer with real shotgun datasets, including RNA-Seq data from 16 CRC libraries (RL Warren, DJ Freeman, P Watson, RA Moore, EA Allen-Vercoe, RA Holt, manuscript submitted), WGS and RNA-Seq from four lymphoma libraries [17] and exon capture data from three ovarian cancer libraries (Figure 2; Additional file 1). HLA predictions were compared to results from these same subjects obtained from standard PCR and capillary sequence-based typing [20]. Results mirrored those obtained from simulated data. For all data types, prediction of allele groups was more reliable than prediction of individual protein coding alleles. For prediction of allele groups, the CRC RNA-Seq data yielded predictions with highest sensitivity and specificity (>96.5%) and low ambiguity (<2.4%), even at low sequence depth (approximately 5 million pairs per sample). From a total of 81 HLA allele groups predicted by HLAminer on the CRC cohort shotgun data only a single allele group prediction conflicted with PCR-based typing results (Additional file 2). For WGS and exome data, high sensitivity and specificity could also be achieved, but only at much higher depth of coverage. For all data types, the ambiguity associated with prediction of individual protein coding alleles were higher than for prediction of allele groups, with predictions from exome data sets more significantly impacted than predictions from WGS or RNA-Seq data sets. HLAminer predictions were also benchmarked on low-coverage 100-nucleotide WGS data from 20 individuals of the 1000 Genomes cohort [15]. HLA class I allele predictions obtained from these same HapMap samples by the targeted PCR method of Erlich and colleagues have been previously published [9]. Applying HLAminer to this data set, allele group sensitivity and specificity of 86.7 ± 15.9% and 86.3 ± 16.1% were achieved (Additional file 3), despite the relatively low number of genome shotgun reads processed (mean ± standard deviation of 361.2 ± 80.9 million. Further, our results from these 1000 Genomes samples are consistent with those we obtained from the diffuse large B cell lymphoma control normal tissue (sensitivity and specificity of 68.8 ± 31.5% and 71.3 ± 21.8%) and tumor tissue (sensitivity and specificity of 75.0 ± 20.4% and 87.5 ± 14.4%) WGS datasets, for which substantially higher sequence coverage was available (approximately 1.1 billion reads per sample). As discussed, the data type availability (WGS) and the lower depth of coverage (10- to 20-fold) are both limiting factors for HLAminer predictions.

HLAminer can evaluate reads by direct alignment (Figure 1, right). However, with Illumina read lengths currently ranging from 100 to 150 nucleotides, this approach has limited utility at the present time. At best we observed 80.0 ± 3.5% sensitivity and 78.2 ± 2.8% specificity (mean ± standard deviation; Figure 2, top panel; Additional file 1).

Regardless of input data, HLAminer predictions for HLA allele groups (two-digit resolution) are more robust than for HLA protein-coding alleles (four-digit resolution) (Figure 2; Additional file 1). Both the sensitivity and specificity of four-digit allele predictions are reduced relative to their two-digit counterparts, but changes to the ambiguity of predictions are more pronounced. For example, with 5 million × 100-nucleotide simulated RNA-Seq read pairs, four-digit predictions show a 8.9% decrease in sensitivity, 8.2% decrease in specificity, and a 31.9% increase in ambiguity, compared to two-digit predictions. This is because HLA coding alleles often differ by only a single base. In contrast to conventional HLA genotyping methods where sequence analysis is restricted to HLA amplicons, a target of reduced complexity compared to shotgun sequence data, HLAminer interrogates the full diversity of sequence information in whole transcriptome, whole genome or whole exome datasets. Here, single base differences can be more easily missed due to factors such as low or unequally distributed sequence coverage and base errors. Thus, the performance of HLAminer for robust four-digit HLA allele calls is a limitation of the current data sets and performance is expected to improve as sequencing technologies evolve to offer greater accuracy and read length at reduced cost.

## Conclusions

HLAminer is the implementation of a strategy for automated HLA typing directly from NGS data sets. It is a fundamentally different approach compared to conventional methods that all rely on first amplifying HLA genes. The identification of allelic variants from an individual NGS data set by simple sequence search or alignment is precluded by the complexity of the locus, the massive allelic diversity in the population and limitations of short sequence reads to adequately capture these variations. The option of typing, retrospectively, existing cohorts for which NGS data have already been generated is enabling, particularly for large community resource projects [12-15]. In this context, the HLA info is value-added, as no additional cost is necessary to generate further HLA specific data from an existing data set. The method can also be applied prospectively. In fact, it may turn out to be the case that it is most efficient to do all HLA typing by shotgun sequencing, since these types of data sets are maximally informative and are becoming routine to generate.

It is recognized that certain HLA allele combinations are common in certain populations, presumably due to linkage disequilibrium [35]. For example the combination of HLA-I A*01; C*07; B*08 is common in some western European populations. These conserved extended haplotypes are not yet well represented in HLA databases, but in future we will explore the possibility of using this type of information to further improve computational HLA typing. We also expect to extend our approach to prediction of HLA class II alleles. HLAminer is available for public use [36].

## Abbreviations

CDS: coding DNA sequence; CRC: colorectal cancer; HLA: human leukocyte antigen; NGS: next-generation sequencing; PCR: polymerase chain reaction; WGS: whole genome shotgun.

## Competing interests

The authors declare that they have no competing interests.

## Authors' contributions

RLW and RAH designed the research; DJF extracted RNA and constructed the sequencing libraries; SM and GC verified predicted HLA calls by conventional sequencing; MC provided ovarian cancer exon capture sequence datasets; RM coordinated the sequencing; RLW and RAH analyzed the data and made the figures; RLW and RAH wrote the manuscript. All authors read and approved the final manuscript.
